# Dermal Alterations in Clinically Unaffected Skin of *Pseudoxanthoma elasticum* Patients

**DOI:** 10.3390/jcm10030500

**Published:** 2021-02-01

**Authors:** Federica Boraldi, Francesco Demetrio Lofaro, Lorena Losi, Daniela Quaglino

**Affiliations:** Department of Life Sciences, University of Modena and Reggio Emilia, Via Campi, 287, 41125 Modena, Italy; francescodemetrio.lofaro@unimore.it (F.D.L.); lorena.losi@unimore.it (L.L.); daniela.quaglino@unimore.it (D.Q.)

**Keywords:** calcification, degradation, elastic fibers, electron microscopy, mesenchymal cells, SMAD

## Abstract

Background: Pseudoxanthoma elasticum (PXE), due to rare sequence variants in the *ABCC6* gene, is characterized by calcification of elastic fibers in several tissues/organs; however, the pathomechanisms have not been completely clarified. Although it is a systemic disorder on a genetic basis, it is not known why not all elastic fibers are calcified in the same patient and even in the same tissue. At present, data on soft connective tissue mineralization derive from studies performed on vascular tissues and/or on clinically affected skin, but there is no information on patients’ clinically unaffected skin. Methods: Skin biopsies from clinically unaffected and affected areas of the same PXE patient (n = 6) and from healthy subjects were investigated by electron microscopy. Immunohistochemistry was performed to evaluate p-SMAD 1/5/8 and p-SMAD 2/3 expression and localization. Results: In clinically unaffected skin, fragmented elastic fibers were prevalent, whereas calcified fibers were only rarely observed at the ultrastructural level. p-SMAD1/5/8 and p-SMAD2/3 were activated in both affected and unaffected skin. Conclusion: These findings further support the concept that fragmentation/degradation is necessary but not sufficient to cause calcification of elastic fibers and that additional local factors (e.g., matrix composition, mechanical forces and mesenchymal cells) contribute to create the pro-osteogenic environment.

## 1. Introduction

Pseudoxanthoma elasticum (PXE, OMIM 264800) is a genetic disease due to rare sequence variants in the *ABCC6* gene [1] that encodes the multidrug resistance-associated protein 6 (MRP6), whose physiological role has not yet been clarified [2]. It has been recently demonstrated that other genes such as *ENPP1* (ectonucleotide pyrophosphatase/phosphodiesterase 1) and *GGCX* (γ-glutamyl carboxylase), in addition to *ABCC6*, have a pathogenic role in calcification-associated diseases (e.g., PXE), as suggested by the occurrence of digenic inheritance [3,4,5].

The disease is typically characterized by fragmentation and calcification of elastic fibers associated to cutaneous, ocular, and vascular manifestations affecting patients’ quality of life [6,7]. The first clinical sign of the disease affects the skin, where laxity and yellow papules appear on flexural body areas (e.g., neck, axilla, groin). Over time, papules may coalesce into large plaques and increased laxity causes loss of skin elasticity, which becomes wrinkled and redundant [2]. Nonetheless, very frequently, cutaneous alterations are misinterpreted, thus delaying the diagnosis. The ocular manifestations, due to calcification of the Bruch’s membrane, have the most dramatic consequences for patients leading to legal blindness. *Peau d’orange*, angioid streaks, and choroidal neovascularization represent the major alterations which are usually described in PXE patients [8,9,10]. The arterial lesions are characterized by mineralized elastic fibers of the medial layer within small and medium-sized arteries [11], and by damages of the endothelial lining. In agreement with these findings, it has been observed an altered balance between circulating mature and circulating progenitor of endothelial cells, indicating vascular damage and low repair potential; moreover, in the blood of PXE patients, an increase of circulating cells with a shift towards the osteoblast-like phenotype has also been demonstrated [12].

PXE is considered a systemic disorder on a genetic basis; however, it has not yet been clarified as to why not all elastic fibers are calcified in the same patient and even in the same tissue. While clinically affected skin has been widely investigated at morphological and biochemical levels, there is no information regarding clinically unaffected skin in the same patient and if the absence of visible alterations is related to normal/abnormal elastic fibers and which are the mechanisms locally controlling mineralization in PXE.

Despite the specific localization of mineral deposits on elastic fibers, by whole exome sequencing, we have demonstrated that the elastin gene did not appear to carry any rare sequence variant; however, changes were observed in a number of genes related to elastic fiber assembly, signaling, and remodeling, suggesting that alterations at the elastin-microfibril interface or at the elastic fiber-cell interface can affect elastic fiber assembly and stability and may influence cell signalling [13].

To date, information on soft connective tissue mineralization comes from studies mainly performed on vascular calcification [14], where it has been demonstrated that cells undergo osteogenic differentiation due to activation of different signalling pathways (i.e., TGF-β signalling, BMPs-Smad-Runx2 signalling, Wnt-Msx2 signalling) [15]. Very recently, the role of TGF-β/BMP signalling has been confirmed in a number of patients with dermal ectopic calcification [13].

Bone morphogenetic proteins (BMPs) belong to the transforming growth factor β (TGF-β) superfamily and regulate a wide spectrum of cellular functions such as cell proliferation, differentiation, and migration. The BMP signalling starts when ligands bind to the BMP receptor complex, that, in turn, phosphorylates cytoplasmic factors such as small mothers against decapentaplegic (Smad)1, 5 and 8. These molecules, together with Smad 4, translocate into the nucleus activating gene transcription factors such as Runx2 (runt-related transcription factor 2), an osteogenic transcription factor [16]. Increased level of phosphorylated-Smad (p-Smad) serves as marker of activation of the BMP signal transduction pathway.

TGF-β is a pleiotropic growth factor secreted as an inactive precursor; after cleavage, mature TGF-β and a latency associated peptide (LAP) form a small latent complex, which binds to large latent TGFβ-binding proteins (LTBP). LTBP interacts with the extracellular matrix, including the elastic fibers. Several proteases such as matrix metalloproteinases (MMP-2 and MMP-9) can cleave LAP and release the mature TGF-β [17]. TGF-β1 binds to serine-threonine protein kinase receptors, which phosphorylate Smad 2 and Smad 3 and together with Smad 4 translocate into the nucleus.

In PXE, increased MMP-2 and MMP-9 activities have been demonstrated, both in vitro and in vivo [18,19]. These proteolytic enzymes cause elastic fibres degradation with the formation of bioactive elastin peptides that actively contribute to vascular calcification [20], but can also release molecules normally sequestered within the extracellular matrix, such as TGF-β [21]. Activation and/or perturbation of the TGF-β signalling pathways plays a key role in the pro-osteoblastic differentiation of dermal mesenchymal cells [22,23]. 

The aim of this study was to investigate clinically unaffected skin of PXE patients focusing on the ultrastructure of elastic fibers and on the p-SMAD1/5/8 and p-SMAD2/3 signalling pathways. 

## 2. Material and Methods

### 2.1. Control Subjects and PXE Patients

In the present study, six skin samples were taken from healthy female subjects as waste tissue resulting from surgical procedures, and from six unrelated female patients of comparable age, who underwent dermal histopathologic diagnosis. In the case of PXE patients, two biopsies were taken: one from affected and the other from clinically unaffected skin (i.e., with or without skin papules and/or laxity). Embedded samples were kept along the years in the laboratory archives. To confirm diagnosis, in five cases, genomic DNA from whole blood was obtained and the search for rare sequence variants in *ABCC6* gene was performed by Sanger sequencing, as previously described [24]. Table 1 shows the PXE causative mutations in the *ABCC6* gene and the severity of the pathological phenotype according to the Phenodex index [25]. In addition to patients reported in Table 1, a skin biopsy was analyzed, for diagnostic purposes, also in an 8-year-old male child exhibiting typical skin papules on the neck and a mild laxity at the axillae. For ethical reasons, we did not perform the biopsy from the non-affected area. Biomolecular analyses of the *ABCC6* gene confirmed clinical diagnosis of PXE (ex12: c.1552C > T, p.Arg518 * and ex30: del30, p.del30).

### 2.2. Ultrastructure Analysis by Transmission Electron Microscopy (TEM)

Samples were fixed in 2.5% glutaraldehyde (Electron Microscopy Sciences, Hatfield, PA, USA) in 0.1 M cacodylate buffer at pH 7.4 for 12 h, post-fixed in 1% osmium tetroxide for 60 min using the same buffer. Dehydrated samples were embedded in Spurr (Electron Microscopy Sciences). Ultrathin sections (60–70 nm) were cut and mounted on 150 mesh copper grids (Electron Microscopy Sciences). Unstained samples were observed with Talos F200S G2 transmission electron microscope (Thermo Fisher Scientific, Waltham, MA, USA).

### 2.3. Immunohistochemistry

To evaluate TGF-β/SMAD signalling pathways, immunohistochemistry was performed on resin embedded skin sections. After treatment in a saturated solution of sodium hydrate in absolute alcohol for 30 min, the sections were incubated at 37 °C for 1 h with p-SMAD1/5/8 antibody (Millipore Corporation, Oakville, CA, USA) and p-SMAD2/3 antibody (Abcam, Cambridge, UK) at 1:50 dilution using the detection kit ultraview DAB (Roche, Monza, Italy) in an automated staining system BenchMark XT (Roche, Monza, Italy), according to the manufacturer’s instructions. Finally, the slides were counterstained with haematoxylin, mounted, and observed with a Zeiss light microscope.

## 3. Results and Discussion

### 3.1. Calcified and/or Degraded Elastic Fibres in Clinically Unaffected Skin

**Figure 1:**
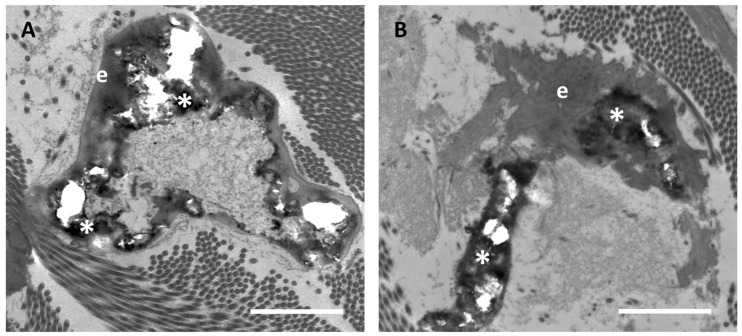
Representative electron microscopy images of calcified elastic fibers which are very rarely observed in clinically unaffected skin (**A**), whereas are highly abundant in the affected (**B**) skin in the same PXE patient. When present, calcification is characterized by electron-dense mineral deposits (asterisk *) altering the elastin amorphous structure (e). Scale bar = 1 µm.

Ultrastructural analyses of clinically unaffected skin (CUS) of patients revealed, although sporadically, calcified elastic fibers (Figure 1A). The rare fibers where mineral deposits were clearly evident were similar to those observed in clinically affected skin (CAS) (Figure 1B). Mineral precipitates were present as heavily electron-dense areas deforming the elastic fibers. Moreover, in calcified fibers, holes can be observed due to loss of mineral deposits during tissue sectioning.

**Figure 2:**
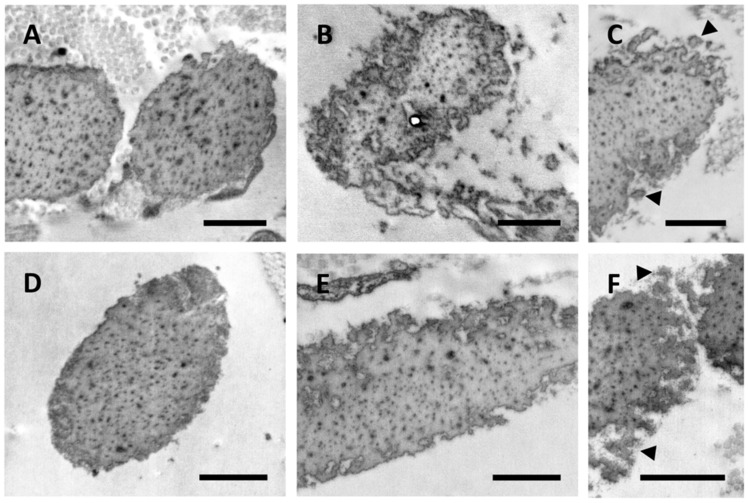
Electron microscopy images obtained from the skin of a 37- and a 52-year-old healthy subject (**A**,**D**, respectively) and from clinically unaffected skin of a 37- and a 50-year-old PXE patient (**B**,**C** and **E**,**F**, respectively). Elastic fiber globules are detached from the main fiber (arrowhead) (**C**,**F**). Scale bar = 1 µm.

Almost all elastic fibers in control samples and in CUS of patients were comprised of an amorphous structure filled with electron dense dots representing matrix constituents, which are known to progressively accumulate within the fibers and affect the elastic properties of the tissue (Figure 2A–F). While, in control subjects, elastic fibers exhibited a regular contour (Figure 2A,D), in the CUS of PXE patients, fibers showed highly irregular and jagged outlines (Figure 2B,C,E,F) with numerous globules detached from the main elastic fiber suggesting the presence of unstable and/or fragmented fibers (Figure 2C,F). These findings support the hypothesis that patients with ectopic calcification exhibit changes in a number of genes affecting packaging, permeability, and/or flexibility of the elastic component, thus weakening elastic fibers’ stability and increasing their susceptibility to proteolysis and, depending on the local context, to calcification [13]. Consistently, these alterations were not related to the patient’s age.

**Figure 3:**
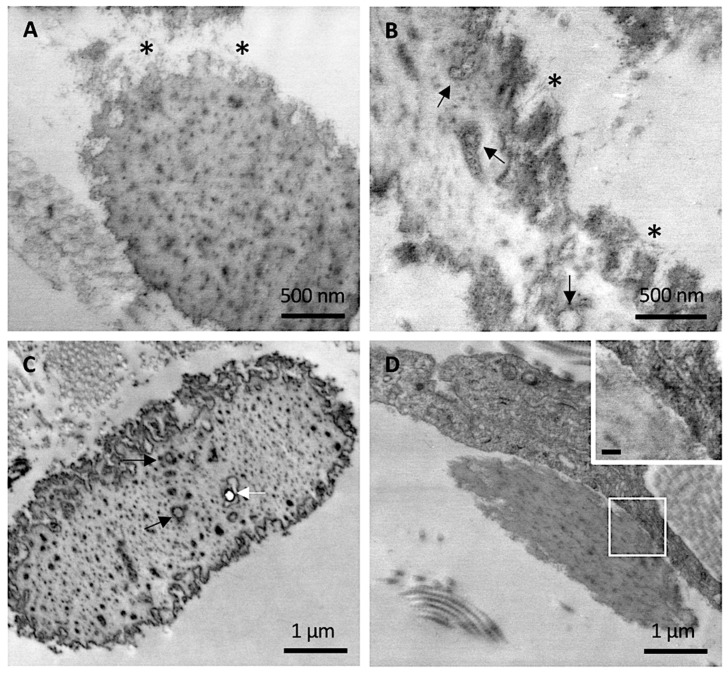
Representative electron microscopy images obtained from clinically unaffected skin. (**A**,**B**) Elastic fibers at the periphery exhibit numerous microfibrillar structures (*) whereas, in the amorphous core, round electron-translucent structures are visible (black arrows). (**C**) Round electron-translucent structures (black arrows) as well as empty holes (white arrow) are visible. (**D**) A dermal fibroblast in close contact with elastic fiber. Insert bar = 100 nm.

At higher magnification, abundant filamentous material was clearly observed at the periphery of mature elastic fibers in CUS (Figure 3A,B), especially in areas where the irregular contour seemed to unveil the microfibrillar scaffold guiding the tropoelastin molecules to form the assembled fiber. Within the amorphous matrix, in addition to the electron-dense deposits, isolated or grouped round electron-translucent structures surrounded by a coat of electron-dense material were observed, possibly representing initial sites of mineral deposition (Figure 3B,C). Consistently, some of these structures were occasionally visualized as small empty holes, suggesting the loss of mineral precipitates during tissue sectioning, as observed for larger crystal deposits in heavily calcified fibers (Figure 3C).

Differently from control skin and from CAS, the fibroblasts in CUS were very frequently in close contact with elastic fibers (Figure 3D).

**Figure 4:**
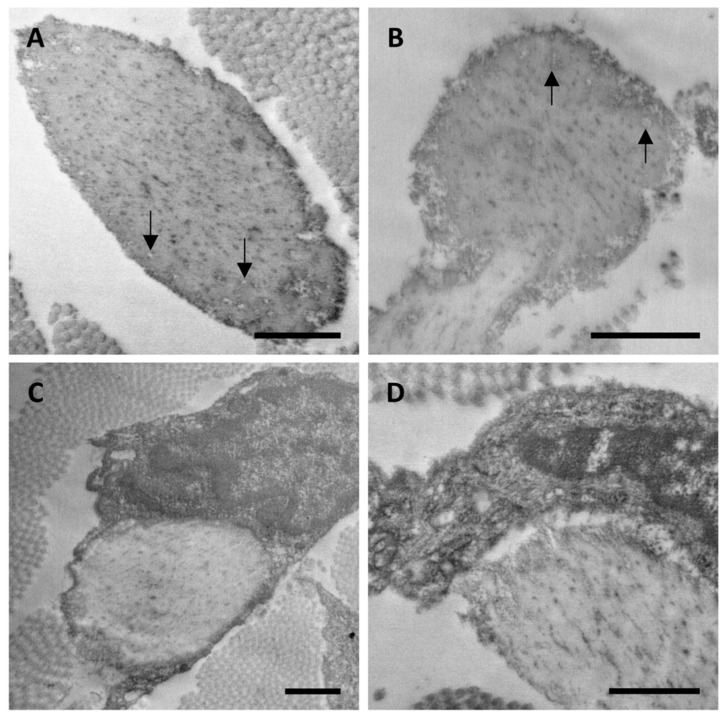
Representative electron microscopy images obtained from clinically affected skin of an eight-year-old child. (**A**,**B**) Filamentous microfibrillar material at the periphery of the elastic fibers and round electron-translucent structures within elastic fibers (arrows), were observed. (**C**,**D**) Fibroblasts embrace and/or are in close contact with elastic fibers. Scale bar = 1 µm.

In agreement with the above described findings, we report ultrastructural data obtained in CAS of an eight-year-old PXE patient. This case has not been included in our experimental design since we did not have the CUS available and therefore it was not possible to compare the CUS and CAS on the same patient. However, results further support the interpretation of data from adult PXE patients. In this young patient, despite the presence of skin laxity in the site of biopsy, we were not able to observe any sign of ectopic calcification. However, elastic fibers exhibit ultrastructural features (i.e., irregular outline and round electron-translucent structures) similar to those observed in CUS of PXE adult patients, although less pronounced (Figure 4A,B). Moreover, many elastic fibers were surrounded (Figure 4C) and/or in close contact with fibroblasts (Figure 4D).

It is known that mesenchymal cells interact and adhere to elastic fibers, this mechanism being very important during the morphogenesis of elastin-rich tissues (e.g., lung, blood vessels, skin) [26] and, according to some authors, also for the repair of damaged elastic fibers [27]. In healthy adults, where elastin synthesis is minimal and its turnover is very low, contacts between cells and elastic fibers are rare. On the contrary, in CAS of the young patient (where calcified elastic fibers were not detected) and in CUS of adult PXE patients (where rare calcified fibers were occasionally observed even at the ultrastructural level), fibroblasts are frequently very close to elastic fibers (Figure 3 and Figure 4). Interestingly, these interactions are less evident in CAS of adult patients characterized by a high number of calcified elastic fibers. The interpretation of these findings is rather challenging, since fibroblasts can interact with elastic fibers either promoting degradation/fragmentation or repair and therefore may contribute to create a pro-osteogenic environment or, on the contrary, to protect the fibers from mineralization.

In a previous study, it was demonstrated that, in plasma and urine of PXE patients, the concentration of desmosines and isodesmosine (i.e., two cross-link amino acids unique of elastin) was higher than in healthy subjects, indicating active elastin degradation [28]. Circulating elastin peptides could stimulate mesenchymal cells to adhere and to repair elastic fibers or alternatively to further fragment elastic fibers [29,30]. In the blood of PXE patients, compared to control subjects [19], elastolytic matrix metalloproteases (i.e., MMP-2 and MMP-9) were significantly increased. Although it has been demonstrated that MMP-2 and MMP-9 are necessary for elastin calcification [31] and that fragmented elastic fibers are more prone to mineralize [32], these conditions are not sufficient to determine crystal deposition. Consistently, despite the general elastolytic condition, in CUS only few fragmented elastic fibers appear calcified, further highlighting the importance of local factors as it has been recently reported, demonstrating that the local environmental context (e.g., pH, ion types) plays a key role in favoring or not mineralization of fragmented/degraded elastin [33].

Finally, at least in the patients we analyzed, no inflammatory cells were found in either CUS or CAS. This finding is in agreement with previous data where inflammatory cytokines levels were similar between PXE patients and healthy subjects [12].

### 3.2. SMAD Signalling

Several studies have shown that the ectopic calcification is an active process finely regulated and dependent on multiple factors such as cell-matrix interactions, pro- and anti-calcifying molecule ratio, and intracellular signalling cascades. BMPs and TGF-β play a crucial role in promoting pathological calcification [13,34]. Within this context, we focused on p-SMAD1/5/8 and p-SMAD2/3 comparing samples of control subjects with skin biopsies from CUS and CAS of the same patient in order to exclude differences due to genetic and/or environmental factors (e.g., diet, smoking).

#### 3.2.1. p-SMAD1/5/8 Signalling Is Active in Clinically Unaffected and Affected Skin

**Figure 5:**
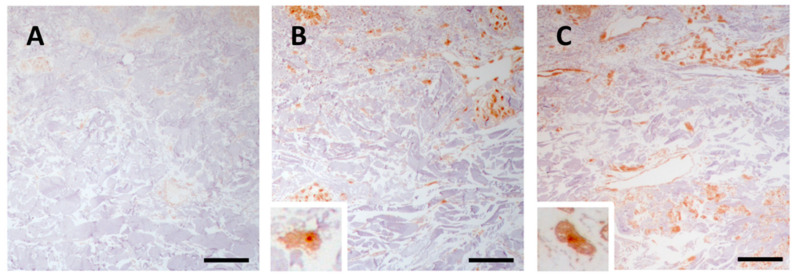
Representative images of immunohistochemistry for p-SMAD1/5/8 in skin samples obtained from the control subject (**A**) and from clinically unaffected (**B**) and affected (**C**) areas in the same PXE patient. Scale bar = 50 μm.

In control skin, there was a weak immunoreactivity for p-SMAD 1/5/8 (Figure 5A), whereas in the dermis of PXE patients, regardless of age, mesenchymal cells were strongly positive for p-SMAD 1/5/8 both in CUS and CAS (Figure 5B and Figure 5C, respectively).

Previous studies have demonstrated that p-Smad1/5/8 was higher in Abcc6 knockout mice compared to control animals [35] and that the p-SMAD1/5/8 signalling was similarly activated in CAS of PXE patients, although images revealed an immunoreaction confined to the mid-dermis and co-localized with mineralization [23]. Our data are in line with previous results obtained in patients with dermal ectopic calcification [13] and demonstrate the intracellular immunolocalization of p-SMAD1/5/8 not only in CAS but also in CUS.

BMP signalling is tightly regulated by different intra- and extra-cellular modulators. For example, reactive oxygen species (ROS) activate BMP signalling in the vascular wall [36]. PXE is characterized, both in vitro and in vivo, by an increase of ROS responsible for a mild chronic oxidative stress, protein carbonylation, and lipid peroxidation [37,38,39]. ROS are generated by different sources such as NADPH oxidase, xanthine oxidase, uncoupled endothelial nitric-oxide synthase, and mitochondria. We recently demonstrated in PXE increased mitochondrial ROS associated with mitochondrial dysfunction (e.g., increased mitochondrial membrane potential, altered mitochondrial morphology, low free calcium within mitochondria) [40]. Therefore, the activation of BMP signalling, observed in PXE, may be favored by oxidative stress.

Furthermore, BMP bioavailability is also regulated by extracellular antagonists such as matrix Gla protein (MGP), which can sequester BMP ligands inhibiting BMP signalling. MGP is a strong inhibitor of ectopic calcification also due to its ability of binding to Ca2+ ions if MGP glutamate residues (Glu) are post-translationally modified into γ-carboxyglutamate (Gla) [41]. Gla-MGP regulates the activity of BMP-2 [41,42,43,44,45] influencing calcification and osteogenic differentiation [46,47]. In particular, it has to be mentioned that ectopic mineralization can be promoted or inhibited depending on BMP-2 and MGP levels [45].

We have demonstrated that: (i) MGP is synthesized by human dermal fibroblasts; (ii) Gla-MGP is significantly lower in PXE than in control cells; iii) in the dermis, Gla-MGP is more abundant inside the control’s fibers than within the patient’s elastic fibers [48]. Thus, in PXE, MGP, being poorly γ-carboxylated, cannot acquire the Ca^2+^-induced conformation necessary for BMP-2 binding and inhibition.

The altered redox balance and the reduced carboxylation of MGP may contribute to the activation of p-SMAD1/5/8 which, in turn, regulates the expression of pro-calcifying genes, such as alkaline phosphatase (ALP/TNAP), an enzyme capable of hydrolyzing pyrophosphate (i.e., inhibitor of ectopic calcification) into inorganic phosphate (i.e., promoter of mineralization) [23,44]. In several studies, it has been highlighted that TNAP is significantly increased in PXE compared to healthy subjects [49,50] and that its up-regulation can be, at least in part, related to the activation of the BMP/SMAD signalling pathway.

#### 3.2.2. p-SMAD 2/3 Signalling Is Active in Clinically Unaffected and Affected Skin

**Figure 6:**
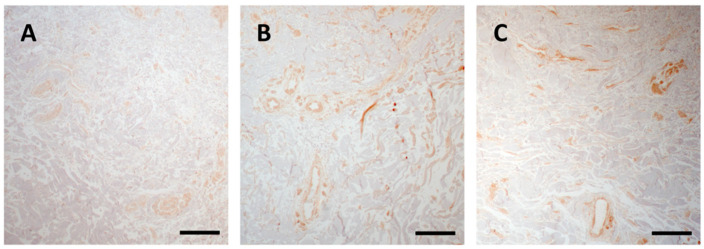
Representative images of immunohistochemistry for p-SMAD2/3 in skin samples obtained from the control subject (**A**) and from clinically unaffected (**B**) and affected (**C**) area in the same PXE patient. Scale bar = 50 μm.

Immunohistochemical analysis revealed that in PXE, regardless of the presence of clinically detectable skin lesions, there was an activation of the TGF-β-SMAD2/3 pathway (Figure 6B,C) if compared to control samples (Figure 6A). Interestingly, in both CUS and CAS, positivity was also observed in cells associated to blood vessels.

It has been reported that TGF-β1/Smad2/3 signalling pathway is involved in the progression of vascular calcification [51] and that accumulation of p-SMAD2/3 proteins is present in the wall of calcified vessels [52].

A positive feedback has been observed between TGF-β and altered redox balance, due to increased ROS and/or to decreased antioxidant systems. In fact, TGF-β can directly promote ROS production or down-regulate the antioxidant systems, and ROS can, in turn, influence TGF-β signalling increasing its expression [53].

TGF-β, upon MMP-dependent cleavage, can activate SMAD signalling and induce osteoblast phenotype transition. MMPs, in fact, not only degrade elastin, but also elastin-associated microfibrils [54] and it is worth mentioning that microfibrils contain latent TGF-β binding proteins (LTBP) [55,56] and, consequently, their degradation determines a release of TGF-β1 [57]. Moreover, elastin-derived peptides have been shown to be chemotactic for different cell types (e.g., fibroblasts, smooth muscle cells) [58] and their binding to cell membrane receptors upregulates MMPs expression and activity [54,59].

Therefore, TGF-β-SMAD2/3 activation in PXE is, at least in part, related to oxidative stress [37,40] and to MMPs activity [18,19].

## 4. Conclusions

Previous investigations have demonstrated that mineral deposition in PXE is specifically localized on the elastic component [6] and it has been suggested that elastic fibers may be less stable and more prone to degradation and possibly to calcification [13,33]. At present, however, it is not known if connective tissue is also involved in clinically unaffected areas.

The main strength of this study is to have analyzed clinically affected and unaffected skin within the same PXE patients in order to focus on the elastic component and on the SMAD-signalling.

Ultrastructural and immunohistochemical analyses indicate that: (i) TGF-β and BMP signalling are similarly activated in both affected and unaffected skin compared to control samples; (ii) in clinically unaffected skin, the majority of elastic fibers are markedly fragmented, whereas calcification is a rare finding occasionally detected at the ultrastructural level in contrast to the abundant calcification typical of the affected skin; (iii) elastin degradation can represent the initial step of elastic fiber calcification, as revealed by ultrastructural observations; (iv) the fragmentation/degradation process is necessary, but not sufficient to induce mineralization of elastic fibers. Therefore, other factors, such as local ion concentration, mechanical stress, and matrix composition [33], through the active involvement of mesenchymal cells [13], have to contribute to create the appropriate pro-osteogenic environment, despite the genetic nature of ectopic calcification in PXE.

## Figures and Tables

**Table 1:** Clinical data of PXE patients (ID) based on Phenodex index and *ABCC6* causative mutations.

ID	Age	Rare Sequence Variant in *ABCC6* Gene		Phenodex Index *		
		Location/Nucleotide/ Amino Acid Variation	Location/Nucleotide/ Amino Acid Variation	Skin	Eye	Cardiac + Vascular
#1	22	Ex9_c.1132C > T; p.Gln378 *	Ex14_c.1798C > T; p.Arg600Cys	1	2	1 + 2
#2	37	IVS17_c.2248-2-1delAG; splicing	Ex23_c.3088C > T; p.Arg1030 *	2	2	1 + 2
#3	50	No blood available		2	2	?
#4	52	Ex19_c.2458G > C; p.Ala820Pro	IVS26_c.3736-1G > A; splicing	1	3	0
#5	53	Ex24_c.3421C > T; p.Arg1141 *	Ex24_c.3421C > T; p.Arg1141 *	3	3	1 + 2
#6	58	Ex12_c.1553G > A; p.Arg518Gln	Ex24_c.3421C > T; p.Arg1141 *	3	3	0

* Skin score: 1 (papules or bumps), 2 (plaques of coalesced papules) and 3 (marked laxity); Eye score: 1 (peau d’orange); 2 (angioid streaks) and 3 (bleeding/scar); Cardiac score: 1 (chest pain/angina/abnormal ECG), 2 (heart attack); Vascular score: 1 (weak or absent pulses), 2 (intermittent claudication), 3 (vascular surgery).

## Data Availability

Not applicable.
